# Characterization of α-isopropylmalate synthases containing different copy numbers of tandem repeats in *Mycobacterium tuberculosis*

**DOI:** 10.1186/1471-2180-9-122

**Published:** 2009-06-09

**Authors:** Wandee Yindeeyoungyeon, Supaporn Likitvivatanavong, Prasit Palittapongarnpim

**Affiliations:** 1 National Center for Genetic Engineering and Biotechnology, NSTDA, Pathumthani 12120, Thailand; 2 Department of Microbiology, Mahidol University, Bangkok 10400, Thailand

## Abstract

**Background:**

Alpha-isopropylmalate synthase (α-IPMS) is the key enzyme that catalyzes the first committed step in the leucine biosynthetic pathway. The gene encoding α-IPMS in *Mycobacterium tuberculosis*, *leuA*, is polymorphic due to the insertion of 57-bp repeat units referred to as Variable Number of Tandem Repeats (VNTR). The role of the VNTR found within the *M. tuberculosis *genome is unclear. To investigate the role of the VNTR in *leuA*, we compared two α-IPMS proteins with different numbers of amino acid repeats, one with two copies and the other with 14 copies. We have cloned *leuA *with 14 copies of the repeat units into the pET15b expression vector with a His_6_-tag at the N-terminus, as was previously done for the *leuA *gene with two copies of the repeat units.

**Results:**

The recombinant His_6_-α-IPMS proteins with two and 14 copies (α-IPMS-2CR and α-IPMS-14CR, respectively) of the repeat units were purified by immobilized metal ion affinity chromatography and gel filtration. Both enzymes were found to be dimers by gel filtration. Both enzymes work well at pH values of 7–8.5 and temperatures of 37–42°C. However, α-IPMS-14CR tolerates pH values and temperatures outside of this range better than α-IPMS-2CR does. α-IPMS-14CR has higher affinity than α-IPMS-2CR for the two substrates, α-ketoisovalerate and acetyl CoA. Furthermore, α-IPMS-2CR was feedback inhibited by the end product l-leucine, whereas α-IPMS-14CR was not.

**Conclusion:**

The differences in the kinetic properties and the l-leucine feedback inhibition between the two *M. tuberculosis *α-IPMS proteins containing low and high numbers of VNTR indicate that a large VNTR insertion affects protein structure and function. Demonstration of l-leucine binding to α-IPMS-14CR would confirm whether or not α-IPMS-14CR responds to end-product feedback inhibition.

## Background

The biosynthesis pathways of the branched-chain amino acids (valine, isoleucine and leucine) all begin with the same precursors (pyruvate or pyruvate and 2-ketobutyrate) and are catalyzed by acetohydroxy acid synthase (AHAS; EC 4.1.3.8). The pathways that lead to valine and isoleucine production have four common enzymatic steps. Leucine biosynthesis via the isopropylmalate (IPM) pathway branches from the valine biosynthesis pathway with the conversion of 2-ketoisovalerate and acetyl CoA to α-isopropylmalate. This first committed step of leucine biosynthesis is catalyzed by α-isopropylmalate synthase (α-IPMS; EC 4.1.3.12). The subsequent two steps are catalyzed by isopropylmalate dehydratase and isopropylmalate dehydrogenase. The final step in the production of leucine is catalyzed by an amino transferase enzyme. The IPM pathway may be the primary metabolic route for producing leucine in bacteria, as enzymes in this pathway have been identified in diverse groups of bacteria [1]. The key enzyme of this pathway, α-IPMS, has been isolated and characterized in bacteria [2-4], fungi [5,6] and plants [7,8]. A comparison of α-IPMS from different species shows that there are significant sequence similarities, suggesting that this enzyme is highly conserved [9].

The *Mycobacterium tuberculosis *genome contains several types of repetitive DNA sequences, including an insertion sequence (IS*6110*), Variable Number of Tandem Repeats (VNTR) [10-13], mycobacterial interspersed repetitive units (MIRU) [12], polymorphic GC-rich repetitive sequences (PGRS) and direct repeats (DR) [14]. Although the polymorphisms of these repetitive sequences have been studied extensively, most of these studies were focused on strain discrimination and epidemiological studies of *M. tuberculosis*. At present, the role of VNTR in *M. tuberculosis *is not well understood.

A VNTR locus, designated VNTR4155, has been found within the coding region of the *leuA *gene. The locus contains repeat units of 57 bp and an extra 9 bp and is polymorphic in various clinical isolates. Repeat units range from 2–21 copies, and those with two copies are the most abundant [15]. The *leuA *gene from most Beijing strains and from the two completely sequenced virulent strains H37Rv and CDC1551 contain two copies of 57-bp tandem repeats. Since the repeats are multiples of 3 bp, a deletion or insertion of 57 bp would not interfere with the translational frame of the protein, but would be result in the deletion or insertion of the repetitive 19-amino acid residues. In fact, deletion of the two 57-bp repeat units seemed to have no effect on the functionality of the mutant α-IPMS compared to the wild-type α-IPMS. This suggests that the repetitive 19-amino acid residues are dispensable [16].

Previously, recombinant α-IPMS from *M. tuberculosis *H37Rv was purified and characterized [4]. A recent investigation reported the kinetics of the enzyme with two copies of the repeat [17]. The three-dimensional crystal structure of α-IPMS has also been solved and shows that Zn^2+ ^and α-KIV bind at the active site, while l-leucine (end product of the pathway that exhibits feedback inhibition to α-IPMS) binds at the regulatory region [18]. The feedback inhibition of α-IPMS by l-leucine is reversible and is described as being a slow-onset inhibition. First, the binding of l-leucine to the enzyme substrate complex causes an inhibitory signal that can be transmitted through the linker domains. A slow isomerization step then occurs, generating a more tightly bound form [19].

It has been shown that *M. tuberculosis *strains that have α-IPMS with three, four and six copies of the repeat units contain proteins of corresponding sizes that can be detected by polyclonal antibodies against α-IPMS [4]. However, it is not known if the *leuA *from *M. tuberculosis *strains that contain very higher numbers of the repeats is translated into a full-length, intact protein with the same activity. In this study, we have cloned, expressed and characterized the products of the *leuA *genes with either two or 14 copies of VNTR. Our results indicate that some enzymatic properties of the recombinant His_6_-tagged α-IPMS with 14 copies of repeats (α-IPMS-14CR) are different from those with two copies (α-IPMS-2CR).

## Results

### Cloning and expression of the *leuA *gene with 14 copies of tandem repeats

**Figure 1:**
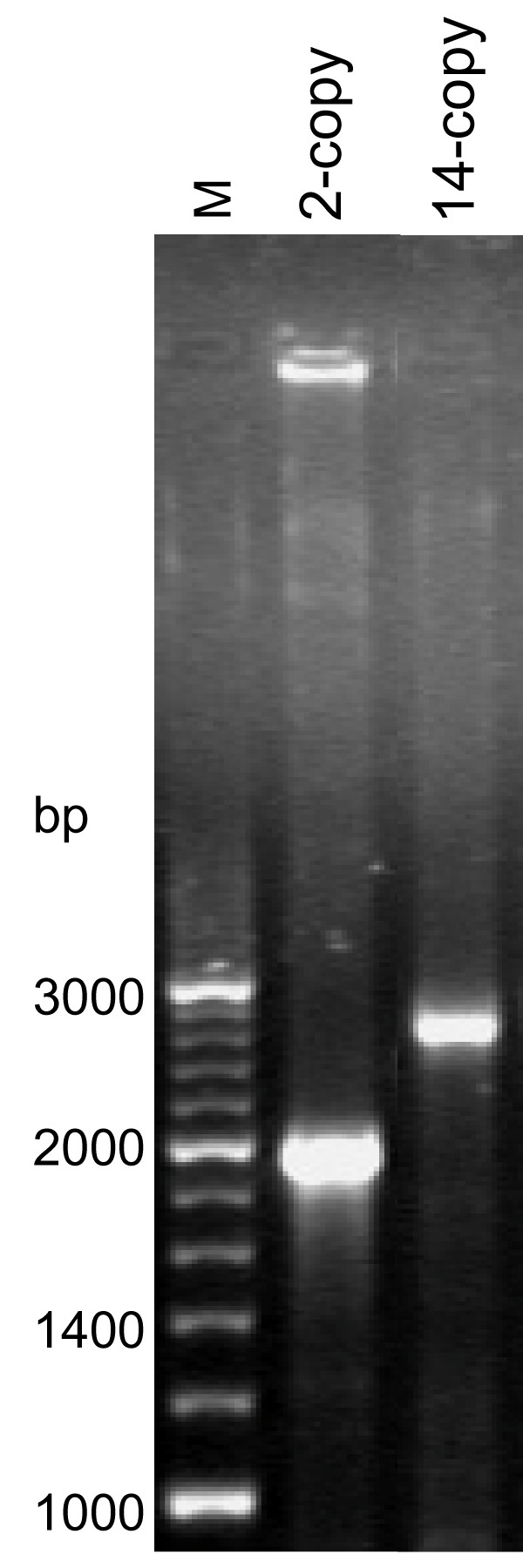
**PCR amplification of *leuA *genes from *M. tuberculosis *strains**. *leuA *genes were PCR amplified from H37Rv and Amnatchareon strain 731 with two and 14 copies of the tandem repeats, respectively. Lane M, 200 bp DNA markers.

**Figure 2:**
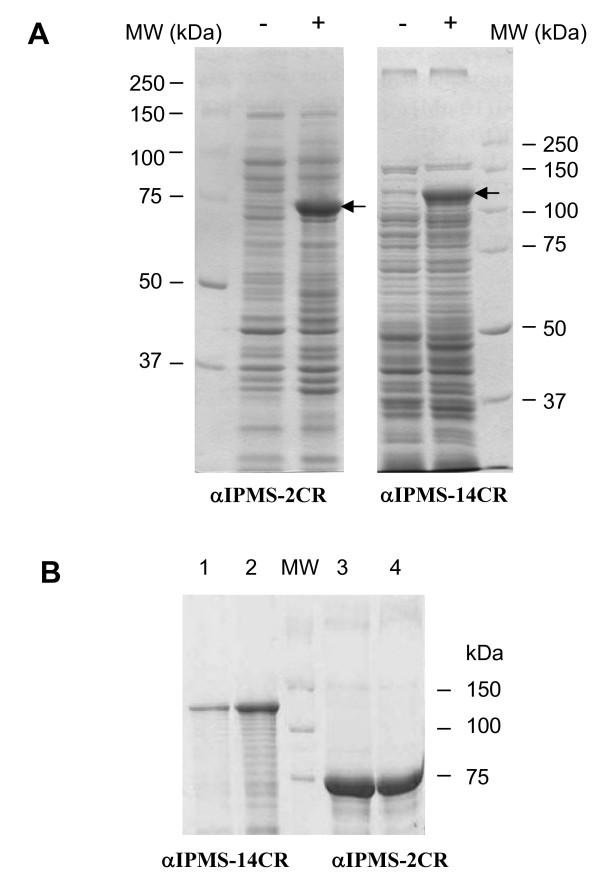
**Analysis of His_6_-α-IPMS proteins on SDS-PAGE**. A) Crude protein extracts of *E. coli *BL21 harboring p2C (α-IPMS-2CR) and p14C (α-IPMS-14CR). Cells were grown overnight at 20°C without (-) or with (+) 0.5 mM IPTG. The cell pellets were sonicated, and the clear lysates were analyzed on 10% SDS-PAGE. Arrowheads indicate protein bands that were induced with IPTG. B) Purified His_6_-α-IPMS proteins from Ni-NTA agarose column. Lanes 1 and 2, elution fractions of His_6_-α-IPMS-14CR; Lane 3 and 4, elution fractions of His_6_-α-IPMS-2CR. MW, molecular weight markers.

The *leuA *gene from *M. tuberculosis *strain 731 contains 14 copies of the VNTR repeat unit and is 2619 bp long. The amplification of *leuA *with the designed primers resulted in PCR products of the predicted size, as shown in Figure 1. DNA sequencing confirmed the copy number of the 57-bp repeat. The amplified DNA fragment of *leuA *with 14 copies of the VNTR repeat unit was cloned into the pET15b expression vector with the N-terminus fused to hexa-histidine (His_6_) in the same fashion as *leuA *from the H37Rv strain, which contains two copies of the repeat unit. The recombinant plasmids, designated p14C and p2C, respectively, were expressed in *E. coli *BL21 (λDE3). The sizes of α-IPMS-2CR and α-IPMS-14CR were the expected sizes (74 and 99 kDa, respectively) of the full-length proteins (Figure 2). We found that induction of enzyme expression by IPTG at low temperature (20°C) results in higher solubility than induction at 37°C. This last condition was critical for α-IPMS-14CR, as it is expressed to lower levels than α-IPMS-2CR. When expressed at 37°C, almost all of the α-IPMS-14CR protein aggregates (i.e., is associated with an insoluble fraction, as assessed by SDS-PAGE (data not shown)).

### Purification of His_6_-tagged proteins under native conditions

The purification of the His_6_-tagged proteins of α-IPMS-2CR and α-IPMS-14CR under native conditions using a Ni-NTA column yielded 90% and 80% pure protein, respectively. These proteins were further purified by gel filtration to approximately 99% purity. The yield of recombinant protein per gram of cell wet weight was 0.4–0.5 mg for α-IPMS-2CR and 0.1–0.2 mg for α-IPMS-14CR. The oligomeric state of each recombinant protein, as suggested by gel filtration analysis, was of a dimer (gel filtration profiles are presented in Additional file 1 and Additional file 2). Although purified α-IPMS-2CR was composed of both dimeric and tetrameric forms, the majority of the protein is in present as a dimer. In addition, the enzymatic activity of the dimeric form was three times higher than that of the tetrameric protein (data not shown). The majority of purified α-IPMS-14CR was in dimeric form, with enzymatic activity six times higher than that of the minor fractions in monomeric form (data not shown).

### Enzymatic properties of His_6_-α-IPMS

**Figure 3:**
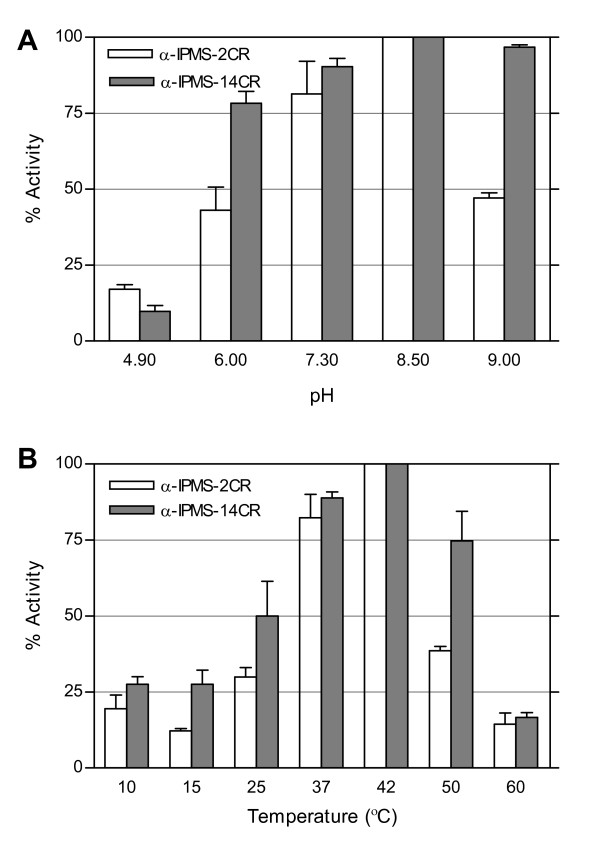
**Activities of His_6_-α-IPMS-2CR and His_6_-α-IPMS-14CR**. Assays were performed as described in the Materials and Methods. Each point is the average of three assays and the vertical bars represent the standard deviations. A) Activities at various pH values at 37°C. B) Activities at various temperatures at pH 8.5.

Both α-IPMS-2CR and α-IPMS-14CR enzymes worked well at a pH between 7.5 and 8.5. At pH 9, α-IPMS-2CR lost much of its activity, while the activity of α-IPMS-14CR remained (Figure 3 panel A). The optimal temperature for both enzymes was approximately 37–42°C. At 50°C, the activity of α-IPMS-14CR remained at 75%, whereas the activity of α-IPMS-2CR dropped below 50% (Figure 3 panel B).

The kinetic parameters of α-IPMS-2CR and α-IPMS-14CR for both substrates are summarized in Table 1. The apparent K_m _and V_max _of α-IPMS-2CR do not agree with those reported previously (K_m _and V_max _for α-ketoisovaleric acid was 24.6 μM and 0.8 U/mg, respectively; K_m _and V_max _for acetyl CoA were 243.5 μM and 2.07 U/mg, respectively) [4]. The reason for these discrepancies is unclear, but may be at least partially due to differences in enzyme preparation and storage conditions. In the previous report, the enzyme was maintained in an elution buffer containing 100–250 mM imidazol, while in this report, dialysis was performed to eliminate imidazol from the enzyme solutions and purified protein fractions obtained by gel filtration were used in the assays.

**Table 1:** Kinetic parameters, V_max _and K_m_, of α-IPMS reacting to α-ketoisovaleric acid and acetyl CoA^*a*^

α-IPMS	α-Ketoisovaleric acid					Acetyl CoA				
	
	K_m_ (μM)	V_max_ (U/mg protein)	R^2^	k_*cat*_^*b*^ (s^-1^)	k_*cat*_/K_m_ (s^-1 ^M^-1^)	K_m_ (μM)	V_max_ (U/mg protein)	R^2^	k_*cat*_^*b*^ (s^-1^)	k_*cat*_/K_m_ (s^-1 ^M^-1^)
α-IPMS-2CR	261 (S.E. = 14.7)	0.49 (S.E. = 0.01)	0.99	1.17	4480	568 (S.E. = 94.5)	0.93 (S.E. = 0.06)	0.99	2.22	3,900

α-IPMS-14CR	35 (S.E. = 5.4)	0.16 (S.E. = 0.01)	0.96	0.52	14,800	27 (S.E. = 6.9)	0.19 (S.E. = 0.01)	0.93	0.61	22,590

^a ^At pH 8.5 and 37°C. The apparent K_m _and V_max _values were determined by varying the concentrations of one substrate at a fixed saturating concentration of the other substrate. α-ketoisovaleric acid and acetyl CoA was fixed at 2 mM and 0.8 mM, respectively. Data are the average of two assays. Prism software (version 3.08) was used for nonlinear regression, curve fit analysis to calculate K_m _and V_max_.

^*b*^k_*cat *_= V_max_/[E] (μmol s^-1^mg^-1^)/(mol mg^-1^)

**Figure 4:**
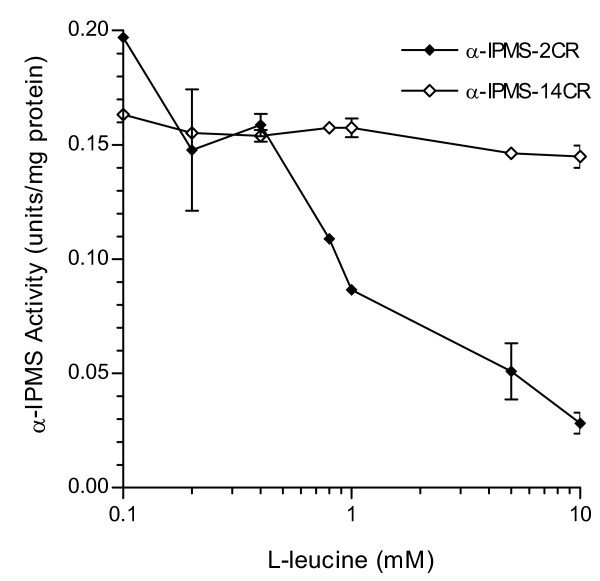
**Inhibition of His_6_-α-IPMS activity by l-leucine**. Activity of His_6_-α-IPMS-2CR and of His_6_-α-IPMS-14CR in the presence of 0.1–10.0 mM of l-leucine. Assays were performed as described in the Materials and Methods at 37°C, pH 8.5. Each point is the average of three assays and the vertical bars represent the standard deviations.

Comparison of the apparent K_m_/V_max _of α-IPMS-2CR and α-IPMS-14CR, processed through similar conditions, shows that α-IPMS-2CR has a lower affinity for its substrates than α-IPMS-14CR (4-fold lower for α-ketoisovaleric acid and 14-fold lower for acetyl CoA). The V_max _values for both substrates of α-IPMS-2CR were higher than those of α-IPMS-14CR, resulting in a higher k_*cat*_. α-IPMS-14CR has a higher catalytic efficiency, however, as k_*cat*_/K_m _ratios for α-ketoisovaleric acid and acetyl CoA were approximately 2 and 5 times higher, respectively, than those of α-IPMS-2CR. The l-leucine feedback inhibition of α-IPMS was investigated with the addition of 0.1 to 10.0 mM l-leucine to the enzyme assay mixtures. The inhibition of α-IPMS-2CR was clearly detectable in the presence of 0.4 mM l-leucine, and the enzyme was inhibited by almost 50% with 0.8 mM l-leucine. l-leucine had no significant effect on α-IPMS-14CR activity under similar assay conditions (Figure 4).

## Discussion

The structure of the *M. tuberculosis *α-IPMS monomer (644 residues) consists of an N-terminal catalytic domain and a C-terminal regulatory domain, which are linked by two small subdomains. The N-terminal domain (residues 51–368) forms an (α/β)_8 _TIM barrel that accommodates the active site. Residues 1–50 function in dimerization. In the linker domain, subdomain I (residues 369–424) is composed of α10 and two short β-strands, while subdomain II (residues 434–490) contains α11-α13. The C-terminal regulatory domain (residues 491–644) is composed of two βββα units (β11, β12, β13, α14 and β14, β15, β16, α15) [18]. The function of the repeat sequences within the coding sequence of α-IPMS remains unclear, as this repeat segment (corresponding to residues 575–612 in the C-terminal domain, between β15 and β16) is disordered in the crystal structure [18]. Singh and Bhakuni (2007) demonstrated that although the isolated TIM barrel domain of α-IPMS retains its folded conformation, it has only 12% of the functional activity of the intact enzyme. This result indicates that the C-terminus influences the activity of the enzyme [20]. Here, we show that α-IPMS-2CR and α-IPMS-14CR are both dimers in solution, as has been observed previously with α-IPMS-2CR [4,17]. The differences between the two enzymes in their activities at high pH and temperature and in some of their kinetic parameters indicate that the copy number of the repeat unit does affect the properties of the protein.

The optimal pH for both α-IPMS-2CR and α-IPMS-14CR was between 7.5 and 8.5, similar to those in other organisms. α-IPMS from *S. typhimurium *[2], *S. cerevisiae *[21], *Clostridium *spp and *Bacteroides fragilis *[3] and Arabidopsis [7] have optimal pHs of 8.5, 8.0, 8.0 and 8.5, respectively. The optimal temperature for both α-IPMS-2CR and α-IPMS-14CR enzymes was the same as the physiological temperature of *M. tuberculosis *(37–42°C). Most previous reports assayed enzymes at the physiological temperatures of their respective organisms as well, e.g., 30°C for yeast α-IPMS and 37°C for *S. typhimurium*α-IPMS. The anaerobic bacteria *Clostridium *spp and *Bacteroides fragilis *have higher optimal temperature for α-IPMS, ranging from 37–46°C [3]. The apparent K_m _values for α-IPMS-2CR and α-IPMS-14CR are different from those previously reported [4,17]. A wide range of K_m _values for α-IPMS activity on α-KIV and acetyl CoA have been reported in *M. tuberculosis *[17], *S. typhimurium *[2] and *S. cerevisiae *[21] (12 and 136, 60 and 200, and 16 and 9 μM, respectively).

de Carvalho and Blanchard (2006) previously demonstrated that the kinetic mechanism of α-IPMS in *M. tuberculosis *is a non-rapid, equilibrium random bi-bi and that the chemistry is not a rate-limiting step in the overall reaction. It was suggested that with physiological substrates, slow substrate binding, product dissociation or conformational changes in the enzyme are likely to be the rate-limiting step. This hypothesis is consistent with the fact that α-IPMS-14CR has a lower V_max _relative to α-IPMS-2CR. α-IPMS-14CR, with the additional 12 copies of the repeat units, is ~30% larger than α-IPMS-2CR.

**Figure 5:**
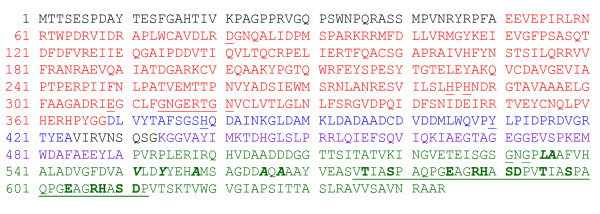
**Amino acid sequence of α-IPMS containing two copies of the VNTR**. The N-terminal domain (catalytic domain), residues 51–368, is colored red. Residues involved in substrate (α-KIV) binding are underlined: D81, H285, H287, N321, E309 and G320. The conserved GxGERxG motif (residues 314–320, H379 and Y410), which forms a groove possible for acetyl CoA binding, is underlined. Linker domain: subdomain I (residues 369–424) is colored blue; subdomain II (residues 434–490) is colored magenta. The C-terminal regulatory domain (residues 491–644) is colored green. The two copies (one copy contains 19 amino acids, vtiaspaqpgeagrhasdp, at residues 575–612) of the repeat sequence are underlined. The hydrophilic and charged residues are in bold. Residues involved in leucine binding are indicated in bold italics: L535, A536, V551, Y554, A565 and A567. Mutation of residues G531, G533 and A536 (underlined) abolished feedback inhibition of α-IPMS in *S. cerevisiae*. The Y410F mutant form of *M. tuberculosis *α-IPMS was insensitive to feedback inhibition.

The lower K_m _(higher affinity for substrates) of α-IPMS-14CR is more difficult to understand. A report on the cystine protease CPB isoforms of *Leishmania mexicana *showed that variation in a few charged amino acid residues located outside of but close to the active site may influence the electrostatic potential on the surface of the proteins, resulting in different K_m _values [22]. In the case of α-IPMS-14CR, although the segment of the protein that includes the 14 copies of the repeat units is located in the C-terminal domain, it may come into close proximity with the active site due to its huge size. The amino acid composition of the repeat units may also be important. Since seven of the 19 residues in the repeat unit are hydrophilic and charged (Figure 5), they could affect the electrostatic potential on the surface of the enzyme and, therefore, the enzyme's affinity for its substrates.

The mechanism of l-leucine inhibition was suggested to be a slow-onset inhibition (time-dependent) [19]. After a rapid formation of an initial inhibitory complex (leucine binds to the regulatory domain), isomerization of the complex occurs, leading to a tightly bound complex. Evidence confirmed that an inhibitory signal is transmitted through the linker domain to the catalytic domain, as the Tyr410Phe mutant form of *M. tuberculosis *α-IPMS is insensitive to l-leucine feedback inhibition [23]. Mutations that abolish l-leucine feedback inhibition in *S. cerevisiae *α-IPMS are clustered around residues surrounding the l-leucine binding site (amino acids Leu-535, Ala-536, Val-551, Tyr-554, Ala-558, Ala565 and Ala-567; Figure 5) [9]. The repeat sequences (residues 575–612) are adjacent to the residues that surround the l-leucine binding site (Figure 5). It is possible that α-IPMS-14CR failed to respond to l-leucine inhibition because the transmission of the l-leucine inhibition signal, the isomerization step or both were obstructed by the large segment of 266 amino acid residues, preventing the formation of the tight complex of enzyme and leucine.

Repetitive DNA sequences can rearrange to increase or decrease the number of the repetitive elements through replication "slippage" events [24]. Thus, strains with low numbers of tandem repeats can evolve to have higher copy numbers and vice versa. VNTR4155 analysis of 85 clinical strains from Amnatchareon showed that the frequencies of bacteria with 2, 3, 10, 11, 14, 16, 17, 18, 19 and 21 copies of the repeat unit are 74.1, 4.7, 7, 1.1, 2.4, 2.4, 2.4, 2.4, 2.4 and 1.1%, respectively [25]. While most strains contain two copies, including most of the Beijing strains, the existence of strains with high copy numbers suggest that there may be a selective advantage to having more repeat units in some environments.

Previous studies have shown that leucine auxotrophs (*leuD*Δ mutants) of *M. bovis *BCG and *M. tuberculosis *are unable to grow in macrophages and in mice [26,27], suggesting that leucine cannot be obtained in such environments. Although there is no data on the amino acid concentrations in *M. tuberculosis *present in macrophages, it can be speculated that α-IPMS proteins with high copy numbers of the repeat may be useful in macrophages. With a lower K_m_, α-IPMS can work sufficiently even at low concentrations of substrate, and with a low V_max_, growth is only partially affected. Moreover, l-leucine feedback inhibition may not be necessary in *M. tuberculosis *when it is residing in macrophages. Whether VNTR4155 contributes to the differential survival in these environments is unknown.

## Conclusion

α-IPMS-2CR and α-IPMS-14CR have significantly different affinities for the two substrates, α-ketoisovalerate and acetyl CoA, and respond differently to inhibition by the enzymatic end-product, l-leucine. The large insertion of the VNTR (14 copies) likely interferes with the enzyme structure and function, though it is also possible that α-IPMS-14CR does not bind l-leucine and, therefore, does not respond to feedback inhibition. Further work on the binding of l-leucine to α-IPMS-14CR will clarify this result.

## Methods

### Materials

Acetyl CoA, DTNB [5,5'-dithio-bis (2-nitrobenzoic acid); Ellman's Reagent], α-ketoisovaleric acid and l-leucine were obtained from Sigma-Aldrich Inc., St. Louis, MO, USA. All other chemicals were obtained from commercial sources and were of reagent grade. Restriction enzymes and T4 DNA ligase were obtained from New England Biolabs, Bevery, MA, USA. *Taq *DNA polymerase was obtained from Invitrogen, Carlsbad, CA, USA.

### Bacterial strains and culture media

*Escherichia coli *strain DH5α was used for maintaining and cloning plasmid DNA. *E. coli *strain BL21 (λDE3) [28] was used for protein expression. *M. tuberculosis *isolate number 731, obtained from a pulmonary tuberculosis patient in Amnatchareon province, Thailand, and the *M. tuberculosis *H37Rv strain (laboratory strain: ATCC 25618) were the sources of the *leuA *gene with 14 and 2 copies, respectively, of the 57 bp tandem repeat [25]. *E. coli *was grown in Luria-Bertani (LB) medium. *M. tuberculosis *was grown on Middlebrook 7H11 agar supplemented with 10% Middlebrook OADC [Oleic acid Albumin Dextrose Catalase] Enrichment (Difco BBL).

### DNA manipulations

Standard protocols for DNA manipulation, DNA transformation, DNA sequencing and PCR amplification were performed as previously described [29,30]. *M. tuberculosis *genomic DNA was prepared as previously described [31].

### Cloning of the *leuA *gene containing 14 copies of the repeat units by PCR amplification

Primer design: two primers, leu44 (5'-GGA ATT CCA TAT GAC AAC TTC TGA ATC GCC C-3') and leu66 (5' -CGC GGA TCC CTA GCG TGC CGC CCG GTT GAC-3') [4], which flank the 5' and 3' ends of the *leuA *gene, were designed to include *Nde*I and *BamH*I recognition sites to facilitate the cloning of the *leuA *gene into pET15b (Novagen). We used 50 μl reaction mixtures containing 50 ng DNA template, 0.2 mM each dNTP, 1 mM each primer, 1.25 mM MgCl_2_, 2 units *Taq *DNA polymerase, 10 mM Tris-HCl (pH 8.3), 50 mM KCl and 0.1% Tween20 for PCR. Reactions were denatured at 94°C for 2 min and then cycled through 30 rounds of denaturation at 94°C for 30 sec, annealing at 62°C for 2 min, and extension at 72°C for 2 min. These cycles were followed with a final cycle at 72°C for 10 min.

PCR products from strain 731 were purified using a PCR purification kit (QIAGEN, Valencia, CA, USA), digested with *Nde*I and *BamH*I, ligated to compatible sites in pET15b and transformed into *E*. *coli *DH5α. Correct clones were identified by colony-PCR and subsequently confirmed by restriction enzyme digestion and DNA sequencing. The PP1 and PP2 primers (PP1: 5'-tac tac gag cac gcg atg a-3', PP2: 5'-GTG ATT GAC GGT GCG AT-3'), which flanked the tandem repeats, were used to sequence the cloned genes. The recombinant plasmids were then transformed into *E. coli *BL21 (λDE3).

### Protein expression

*E. coli *BL21 (λDE3) cells harboring the recombinant plasmids were grown at 37°C in LB medium supplemented with 100 μg/ml of ampicillin until the culture reached mid log phase (~0.3–0.4 OD_600_). IPTG was added to the culture to a final concentration of 0.5 mM. The culture was incubated at 20°C with shaking overnight. The bacterial cells were harvested by centrifugation, washed once with 50 mM Tris-HCl, pH 7.0, and stored at -70°C until use.

### Protein purification

One milligram of cells (wet weight) from 200 ml of culture media was resuspended in 1 ml lysis buffer (10 mM NaH_2_PO_4_, 300 mM NaCl, 10 mM imidazole, pH 8.0) and lysed by sonication. The cell lysate was centrifuged at 10,000 *g *for 30 min to separate the soluble and insoluble fractions. Cleared lysate containing the His_6_-tagged protein was transferred to a tube containing 0.5 ml of 50% Ni-NTA agarose (QIAGEN, Valencia, CA, USA) saturated in lysis buffer. The lysate was mixed with the Ni-NTA resin and incubated at 4°C for 60 min. The mixture was then transferred to a 5 ml column, and the flow-through fraction was collected. The column was washed three times with 5 ml wash buffer (50 mM NaH_2_PO_4_, 300 mM NaCl, 20 mM imidazole, pH 8.0). The recombinant protein was then eluted with 0.5 ml elution buffer (50 mM NaH_2_PO_4_, 300 mM NaCl, 250 mM imidazole, pH 8.0), dialyzed against 50 mM phosphate buffer, pH 7.0, concentrated by Amicon Ultra centrifugal filter (Millipore) and stored in 50% glycerol at -70°C.

### Protein analysis and gel filtration

Protein fractions were analyzed by 10% SDS-PAGE. Protein concentration was determined by the Lowry method [32]. Purified recombinant proteins were applied to a Superdex 200 HR/30 column saturated with 50 mM sodium phosphate, 150 mM NaCl, pH 7.0 (Amersham Pharmacia Biotech, column diameter = 2 cm and column length = 70 cm). A mobility standard curve was constructed from standard markers: vitamin B12 (1.355 kDa), cytochrome C (12.4 kDa), carbonic anhydrase (29.0 kDa), BSA (66.0 kDa), alcohol dehydrogenase (150 kDa) and β-amylase (200 kDa). The column was run at a flow rate of 0.25 ml/min. The volume of each collected fraction was 0.5 ml. Fractions containing proteins were concentrated using an Amicon Ultra centrifugal filter (Millipore). Glycerol was added to a final concentration of 50% before storing at -70°C.

### Enzyme assay

The procedure for analyzing α-IPMS activity is an end-point assay using DTNB [5,5'-dithio-bis (2-nitrobenzoic acid)] to detect the formation of coenzyme A (CoA) at 412 nm (ε = 14140 M^-1^cm^-1^) [2]. Reaction mixtures of 150 μl, containing 50 μmoles Tris-HCl, pH 8.5, 20 μmoles KCl, 0.2 μmoles acetyl CoA and 0.5 μmoles α-ketoisovaleric acid, were pre-incubated to 37°C for five min. The enzyme was added in a volume of 100 μl to the reaction mixtures. After incubating at 37°C for five min, the reaction was stopped with the addition of 0.75 ml absolute ethanol and 0.5 ml 1 mM DTNB. To determine the optimal pH, enzymes were assayed at pH 5, 6, 7, 8.5 and 9 at 37°C. The enzymes were also assayed at pH 8.5 at 10, 15, 25, 37, 42, 50 and 60°C. The K_m _and V_max _kinetic parameters using the two substrates, α-ketoisovaleric acid and acetyl CoA, were determined using highly purified proteins (gel filtration fractions) at pH 8.5 and 37°C. In the assays, the concentration of acetyl CoA was fixed at 0.8 mM, while the concentration of α-ketoisovaleric acid varied from 0.02–2.0 mM or the concentration of α-ketoisovaleric acid was fixed at 2 mM, while the concentration of acetyl CoA were varied from 0.02–1.6 mM. In the product inhibition assay, l-leucine was included in the reaction mixtures at a final concentration of 0.1, 0.2, 0.4, 0.8, 1.0, 5.0 and 10.0 mM.

One unit of enzyme is defined as the amount catalyzing the formation of 1 μmole CoA per minute [33]. Enzyme activity (V) is defined as units (of enzyme) per milligram of protein. The kinetic data were fitted to the Michaelis-Menten equation by a non-linear least square regression method. The calculations and graphic results were generated by Prism 3.03 software. The catalytic constant k_*cat *_= V_max_/[E] (μmol s^-1^mg^-1^)/(mol mg^-1^). The molar concentrations of α-IPMS-2CR and α-IPMS-14CR were 1.426 × 10^-8 ^and 1.084 × 10^-8 ^moles/mg, respectively.

## Authors' contributions

SP generated recombinant plasmids. WY performed the enzyme purification and analysis and drafted the manuscript. PP revised the drafted manuscript. All of the authors read and approved the final version of the manuscript.

## Supplementary Material

Additional file 1 **Gel filtration profiles of α-IPMS-2CR**. Gel filtration of α-IPMS-2CR. Material, Superdex 200 HR/30. A, B, C, D, E, F, and G (with arrows) refer to the peak positions of blue dextran, amylase, alcohol dehydrogenase, BSA, carbonic anhydrase, cytochrome C, and vitamin B12. The major peak fractions was dimer protein and the minor peak fractions was tetramer protein. Enzyme activity of the minor peak fractions was approx. 1/3 of the major peak fractions. Click here for file

Additional file 2 **Gel filtration profiles of α-IPMS-14CR**. Gel filtration of α-IPMS-14CR. Material, Superdex 200 HR/30. A, B, C, D, E, F, and G (with arrows) refer to the peak positions of blue dextran, amylase, alcohol dehydrogenase, BSA, carbonic anhydrase, cytochrome C, and vitamin B12. The major peak fractions was dimer protein and the minor peak fractions was monomer protein. Enzyme activity of the minor peak fractions was approx. 1/6 of the major peak fractions. Click here for file
