# Supplementary figures and images for: Characterization of α-isopropylmalate synthases containing different copy numbers of tandem repeats in Mycobacterium tuberculosis
Source: BMC Microbiol. 2009 Jun 9;9:122. doi: 10.1186/1471-2180-9-122 (PMC2704214; doi:10.1186/1471-2180-9-122)

## Slide 1
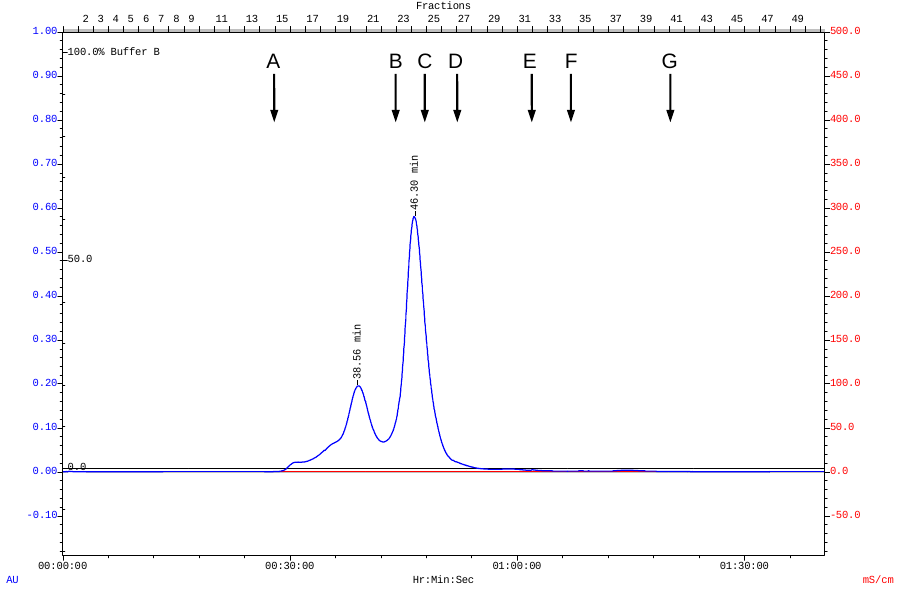

A
B
C
D
E
F
G

Supplement: Additional file 1 — Gel filtration profiles of α-IPMS-2CR. Gel filtration of α-IPMS-2CR. Material, Superdex 200 HR/30. A, B, C, D, E, F, and G (with arrows) refer to the peak positions of blue dextran, amylase, alcohol dehydrogenase, BSA, carbonic anhydrase, cytochrome C, and vitamin B12. The major peak fractions was dimer protein and the minor peak fractions was tetramer protein. Enzyme activity of the minor peak fractions was approx. 1/3 of the major peak fractions. [file 1471-2180-9-122-S1.ppt]

## Slide 1
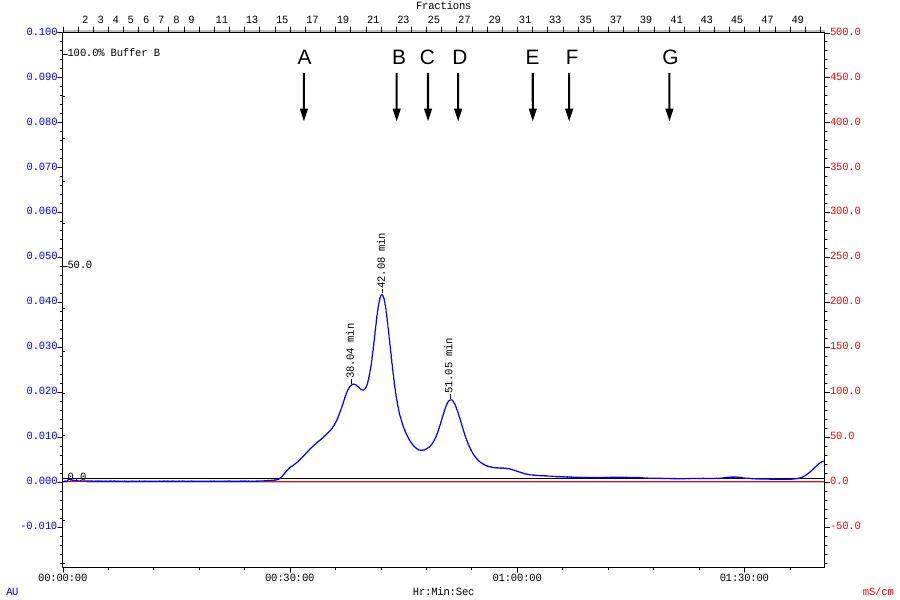

B
D
E
F
G
A
C

Supplement: Additional file 2 — Gel filtration profiles of α-IPMS-14CR. Gel filtration of α-IPMS-14CR. Material, Superdex 200 HR/30. A, B, C, D, E, F, and G (with arrows) refer to the peak positions of blue dextran, amylase, alcohol dehydrogenase, BSA, carbonic anhydrase, cytochrome C, and vitamin B12. The major peak fractions was dimer protein and the minor peak fractions was monomer protein. Enzyme activity of the minor peak fractions was approx. 1/6 of the major peak fractions. [file 1471-2180-9-122-S2.ppt]
